# Serovar distribution, antimicrobial resistance profiles, and PFGE typing of *Salmonella enterica* strains isolated from 2007–2012 in Guangdong, China

**DOI:** 10.1186/1471-2334-14-338

**Published:** 2014-06-17

**Authors:** Bixia Ke, Jiufeng Sun, Dongmei He, Xiaocui Li, Zhaoming Liang, Chang-wen Ke

**Affiliations:** 1 Institute of Microbiology, Guangdong Provincial Center for Disease Control and Prevention, 511430 Guangzhou, China; 2 Guangdong Provincial Institute of Public Health, Guangdong Provincial Center for Disease Control and Prevention, 511430 Guangzhou, China

**Keywords:** *Salmonella enterica*, Non-typhoidal *Salmonella*, Antimicrobial susceptibility, Ciprofloxacin, Cephalosporins, MDR, PFGE

## Abstract

**Background:**

*Salmonella enterica* includes the major serovars associated with human salmonellosis. In this study, 1764 clinical *Salmonella enterica* isolates from diarrhea outpatients were collected from fifteen cities in Guangdong province, China, between 2007 and 2012. These isolates represent all of the *Salmonella* isolates collected from the province during that period.

**Methods:**

The isolates were characterized by serovar determination, antimicrobial susceptibility tests and PFGE fingerprint typing.

**Results:**

The serovar distribution results demonstrated that *Salmonella* Typhimurium (n = 523, 29.65%) and *Salmonella* 4,5,12:i:- (n = 244, 13.83%) are the most common serovars causing infant salmonellosis, whereas *Salmonella* Enteritidis (n = 257, 14.57%) mainly causes human salmonellosis in adults. The serovar shift from *Salmonella* Enteritidis to *Salmonella* Typhimurium occurred in 2008. Antimicrobial susceptibility data showed a high burden of multidrug resistance (MDR) (n = 1128, 56.58%), and a 20%-30% increase in the number of isolates resistant to ciprofloxacin (n = 142, 8.05%) and third-generation cephalosporins (n = 88, 4.99%) from 2007–2012. Only 9.97% of isolates (n = 176) were fully susceptible to all agents tested. A high burden of MDR was observed in *Salmonella* Typhimurium and *Salmonella* 4,5,12:i:- for all age groups, and a reduced susceptibility to third-generation cephalosporins and quinolones occurred particularly in infants (≤6 years). The dominant PFGE patterns were JPXX01.GD0004, JEGX01.GD0006-7 and JNGX01.GD0006-7. ACSSuT was the predominant MDR profile in the *Salmonella* Typhimurium & 4,5,12:i:- complexes, while ASSuT-Nal and ASSu-Nal were the major MDR profiles in *Salmonella* Enteritidis. The predominant PFGE patterns of the *Salmonella* Typhimurium & 4,5,12:i:- complexes and *Salmonella* Stanley were most prevalent in infants (≤6 years). However, no obvious relationship was observed between these PFGE profiles and geographic location.

**Conclusions:**

These data reveal the serovar distribution of isolates recovered from diarrhea patients, the characteristics of resistant strains and fingerprint typing in Guangdong from 2007 to 2012. These results highlight a serovar shift and a worrying percentage of MDR strains with increasing resistance to quinolones and third-generation cephalosporins. Thus, continued surveillance of *Salmonella* and their MDR profiles using combined molecular tools and efforts to control the rapid increase in antimicrobial resistance among *Salmonella* in Guangdong are needed.

## Background

The genus *Salmonella* currently includes two species, *Salmonella enterica* and *Salmonella bongori. Salmonella enterica* is further divided into subspecies I (*enterica*), II (*salamae*), IIIa (*arizonae*), IIIb (*diarizonae*), IV (*houtenae*), and VI (*indica*). Serotyping is the traditional method for the subtyping and the differentiation of *Salmonella* isolates based on the Kauffmann–White scheme. Over 2500 *Salmonella* serotypes are currently known [1]. *Salmonella enterica*, the etiological agent of salmonellosis, is recognized as an endemic food-borne pathogen that causes a heavy global socioeconomic burden [2-4]. *Salmonella enterica* infections result in diverse clinical manifestations. Typhoid fever, caused by *Salmonella* Typhi and *Salmonella* Paratyphi, is a bacteremic illness which occurs in strong, healthy hosts in addition to immunocompromised hosts and infants and is essentially dependent on the bacterial inoculum/infectious dose [5]. Non-typhoidal *Salmonella* (NTS) serovars (e.g., *Salmonella* Enteritidis, *Salmonella* Typhimurium and *Salmonella* 4,5,12:i:-) normally cause self-limiting diarrhea or gastroenteritis, which results in a significant disease burden, with an estimated 2.8 million cases of diarrhea and 93.8 million cases of gastroenteritis, worldwide, and 155,000 deaths each year [6]. The host range of NTS serovars is broad, including poultry and cattle. Infection caused by NTS is commonly due to contamination in the food chain and food poisoning in developing countries. Occasionally, NTS serovars cause secondary bacteremia, while in immunocompromised patients in sub-Saharan Africa, they cause high rates of bacteremia in children below 5 years old as well as those with HIV infection [5,6].

Typhoid *Salmonella* caused 216,000 deaths globally in 2010 [2], whereas most cases of invasive, non-typhoid salmonellosis occur occasionally and are due to either *Salmonella* Typhimurium or *Salmonella* Enteritidis [7]. The resistance of NTS to ampicillin, chloramphenicol and sulphamethoxazole is usually high in Africa and the USA [8,9]. The treatment of NTS, therefore, increasingly relies on fluoroquinolones or third-generation cephalosporins; however, these treatment options are threatened by reduced bacterial susceptibility to fluoroquinolones and extended-spectrum beta-lactam antibiotics, respectively [9,10]. In Hong Kong, China, the rate of infections by NTS has been increasing since 1989, and the most common *Salmonella* serovar is Enteritidis [11]. The antimicrobial susceptibilities of 275 *Salmonella* Enteritidis strains isolated from 1986 to 1996 show that over 99% of the isolates are susceptible to 17 of the 19 antimicrobial agents tested [12]. From 2005 to 2010, *Salmonella* Enteritidis was the predominant serovar in Hong Kong; in that time, the strains that were not susceptible to ciprofloxacin increased significantly, from 39.3% to 63%, and the percentage of multidrug resistant (MDR) strains increased from 17.8% to 36.2% [13]. In an inland province, Henan, the two most common serovars among the isolates collected during 2006 and 2007 were *Salmonella* Typhimurium (27%) and *Salmonella* Enteritidis (17%) [14]. Our previous study on *Salmonella* infection in diarrhea patients in Guangdong province showed that *Salmonella* Typhimurium and Enteritidis were the most common serovars. Eighty percent of the 229 isolates were susceptible to cephalosporins and quinolones, while 59.3% of them were multidrug resistant strains [15-18]. However, these studies did not provide sufficient information concerning prevalence, serovar distribution, and antimicrobial susceptibility in Guangdong province. Reduced quinolone- and third-generation cephalosporin- susceptibility in NTS is also poorly documented [10,19].

The current study describes serovar profiles and potential profile shifts, characterizes MDR isolates with a special focus on susceptibility to quinolones and third-generation cephalosporins and reveals associations between MDR profiles and PFGE fingerprint pattern.

## Methods

### Ethics statement

Ethical approval was granted by the Ethical Committee of the Guangdong Provincial Center for Disease Control and Prevention. The present study complies with the World Health Organization and international guidelines on global surveillance [20]. The data have been reviewed and analyzed anonymously.

### Sampling, bacterial culture and identification

**Figure 1:**
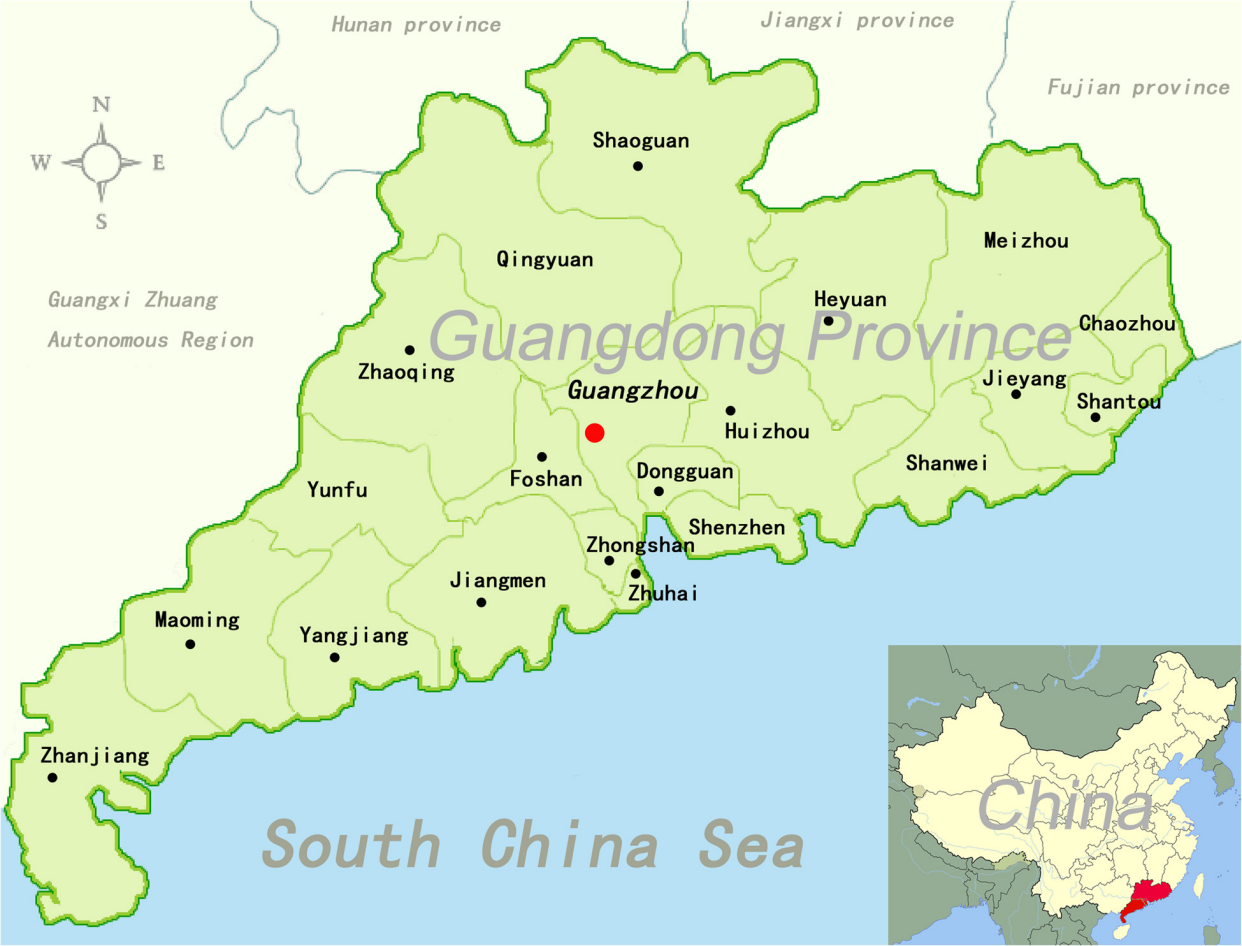
**Locations where clinical diarrhea outpatients collected in Guangdong Province (marked with green color).** The 15 isolation sites are marked in red and black dots in Guangdong Province, China. International borders and location of Guangdong province are also shown in the figure.

According to the Global *Salmonella* Surveillance program, all the cities in Guangdong Province were included in this system (Figure 1), while 15 of 21 cities submitted the samples to our center for *Salmonella* surveillance. The recruitment of patients followed specific guidelines. Patients who had two of the following three symptoms were chosen for the study: 1) diarrhea more than three times within 24 hours, with watery stools; 2) fever > 38°C, headache, chills, malaise; 3) diarrhea with vomiting, abdominal pain, and watery stools. Stool samples were collected from clinical diarrhea outpatients of hospitals in fifteen cities from 2007 to 2012 (Figure 1). All of the samples (42,889) were cultured in local hospitals using solid broth agar media (Huankai, Guangzhou, China) for the initial evaluation of *Salmonella* growth, and then suspicious colonies were picked into vials containing semi-solid Rappaport Vassiliadis Medium (OXOID, France), and shipped to Guangdong Provincial Center for Disease Control and Prevention (Guangzhou, China) at room temperature, where they were incubated daily and checked for growth. The cultures that grew were Gram stained, subcultured and identified at the species level by standard biochemical methods, including the characteristic growth on Kligler Iron Agar (Huankai, Guangzhou, China), tests for urease, oxidase, b-galactosidase and indole production, and positive tests for lysine decarboxylase (the technician training course was first launched on Apr, 2007, and updated on Sep, 2009). In total, 1764 stool samples showed positive results for *Salmonella* growth. The serovar of the *Salmonella* isolates was confirmed by slide agglutination with commercial monospecific anti-sera (Sifin, Berlin, Germany), following the White-Kauffmann-Le Minor scheme [21]. The outbreak isolates were collected separately, and all the strains collected as described above were considered epidemiologically unrelated. Age, gender and geographic origin were recorded as part of the standard information present on the laboratory request forms.

### Antimicrobial susceptibility

The antimicrobial susceptibility test was performed according to the guidelines of the Clinical and Laboratory Standards Institute (CLSI) [22]. The agar diffusion assays were performed on Muller-Hinton agar with disks containing seven classes of antimicrobial agents (aminoglycoside, cephalosporin, penicillin, quinolone, tetracycline, amphenicol, and sulfonamide). The specific antimicrobials used were: streptomycin 10 μg (S), gentamicin 10 μg (G), ceftazidime 30 μg (Caz), cefotaxime 30 μg (Ctx), cefepime 30 μg (Fep), ampicillin 10 μg (A), nalidixic acid 30 μg (Nal), ciprofloxacin 5 μg (Cp), tetracycline 30 μg (T), chloramphenicol 30 μg (C), sulfamethoxazole 300 μg (Su), and trimethoprim 5 μg (Tm). The results were interpreted as resistant, intermediate or susceptible, according to the CLSI guidelines [22]. *Escherichia coli* ATCC25922 was used as quality control organism. MDR was identified as resistance to more than three classes of antimicrobials. The antimicrobial resistance pattern and the sources of the strains are shown in Additional file 1: Table S1.

### Pulsed field gel electrophoresis

The pulsed field gel electrophoresis (PFGE) results were taken from the routine surveillance data in our lab. PFGE was performed according to the PulseNet protocol for *Salmonella*[23], using XbaI as restriction enzyme (New England Biolabs, Leusden, The Netherlands). The cluster analysis was performed with Bionumerics 5.1 (Applied Maths NV, Sint-Martens-Latem, Belgium) using the Dice similarity coefficient and UPMGA (unweighted pair group method using average linkages) dendrogram type (optimization 0.50%, position tolerance 1.50%).

### Data analysis

All data were entered in an Excel database (Microsoft Corporation, Redmond, Washington, USA). Proportions were assessed for statistical significance using the Chi square test or Fisher’s exact test, considering *p <* 0.05 as significant. If the data were normally distributed, the mean values of two groups were compared with Student’s t-test; otherwise, median values were compared with the Mann–Whitney rank sum test (Stata 10, Stat Corp, Texas, USA).

## Results

### Serovars and epidemiological data

**Figure 2:**
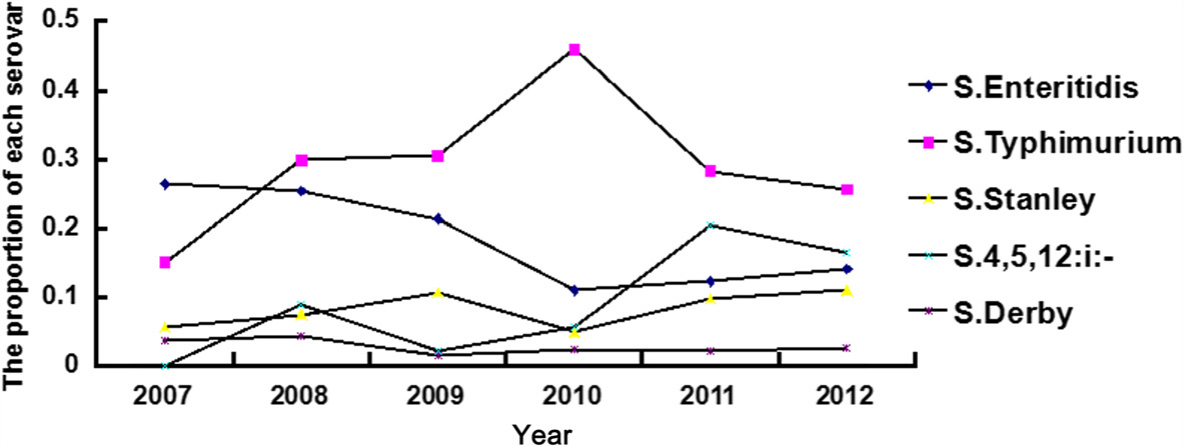
**The proportion of top 5 serovars among the total isolates from 2007 to 2012.** Each data point means the proportion of each serovar in the present year (indicated as percentage in Y-axis), different serovars are indicated using different symbols as illustrated in figure.

In total, 1,764 *Salmonella* isolates were successfully isolated from 42,889 stool samples, which were collected from diarrhea outpatients in 15 cities in Guangdong Province from 2007–2012 (Additional file 1: Table S1). The serovar identification showed that these isolates could be classified into 128 serovars. The most prevalent strains isolated during the test period were NTS (Table 1). The predominant NTS consisted of *Salmonella* Typhimurium (n = 523, 29.65%), *Salmonella* Enteritidis (n = 257, 14.57%), *Salmonella* 4,5,12:i:- (n = 244, 13.83%), *Salmonella* Stanley (n = 167, 9.47%), *Salmonella* Derby (n = 46, 2.61%), *Salmonella* Rissen (n = 36, 2.04%), *Salmonella* Weltevreden (n = 35, 1.98%), *Salmonella* Infantis (n = 28, 1.58%), *Salmonella* Agona (n = 24, 1.36%), *Salmonella* Albany (n = 22, 1.25%) and *Salmonella* Newport (n = 19, 1.08%) (Table 1). The incidence of *Salmonella* infections caused by the top 11 NTS serovars (n = 1401, 79.42%) showed an increasing trend from 2007 to 2012, except for *Salmonella* Typhimurium in 2010 (Table 1). In 2007, *Salmonella* Enteritidis was the major causative serovar in diarrhea outpatients (n = 14, 26.4%), while in 2008, *Salmonella* Typhimurium (n = 20, 29.85%) became more frequently isolated than *Salmonella* Enteritidis (n = 17, 25.30%). From that point, *Salmonella* Typhimurium and *Salmonella* 4,5,12:i:- were the predominant endemic serovars in diarrhea outpatients in Guangdong (Figure 2).

**Table 1:** **Distribution of the predominant serovars of 1764 *Salmonella enterica *** **isolates from humans with infections in Guangdong Province, China from 2007 to 2012**

***Salmonella enterica*** **serovars**	**2007**	**2008**	**2009**	**2010**	**2011**	**2012**	**In total**	**% of total isolates**
Enteritidis	14	17	28	31	48	119	257	14.57
Typhimurium	8	20	40	129	110	216	523	29.65
1,4,5,12:i:-	0	6	4	16	79	139	244	13.83
Stanley	3	5	14	14	38	93	167	9.47
Derby	2	3	2	7	9	23	46	2.61
Rissen	0	0	0	0	12	24	36	2.04
Weltevreden	0	0	2	9	7	17	35	1.98
Infantis	0	0	3	4	12	9	28	1.58
Agona	1	2	4	0	3	14	24	1.36
Albany	0	1	1	5	6	9	22	1.25
Newport	1	0	3	6	0	9	19	1.08
Typhi	0	0	1	1	1	1	4	0.23
Paratyphi(A + B + C)	1 (B)	0	3(B)	5(2A + 2B + 1C)	3(B)	12(3A + 9B)	24	1.36
other 114 NTS serovars	23	14	26	54	61	158	335	18.99
Total	53	68	131	281	389	843	1764	100

**Figure 3:**
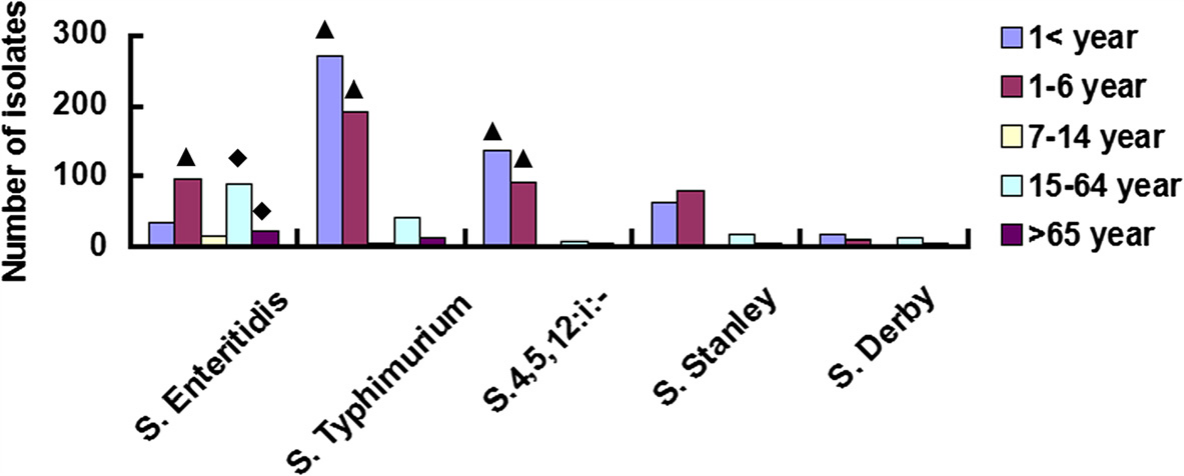
**The prevalence of top 5 serovars among the total isolates within different age groups.** Less than one years old (<1): newborn baby group; between 1 and 6 years old (1–6): Infants group; between 7 and 15 years old (7–14): juvenile; between 15 and 64 years old (7–14): adults group; more than 65 years old (>65): elderly group. The “▲” mark the predominant serovar in less than 6 years old group, and “♦” indicate the most prevalent serovar in adults.

The male/female ratio of the patients was 1.37 (1020 male, 744 female). The age distribution of the patients with the predominant *Salmonella* serovars is illustrated in Figure 3. The median patient age was 0.92 years (interquartile range: 0.58–4 years). The age-associated stratified analysis showed that *Salmonella* Typhimurium and *Salmonella* 4,5,12:i:- were the predominant infectious agents in infants (<6 years), whereas *Salmonella* Enteritidis was the predominant serovar in adults (>15 years). However, there was no statistically significant difference between the median age of *Salmonella* Typhimurium, *Salmonella* 4,5,12:i:- and *Salmonella* Enteritidis patients (*p >* 0.05, Mann–Whitney Rank Sum Test).

### Antimicrobial susceptibility

**Figure 4:**
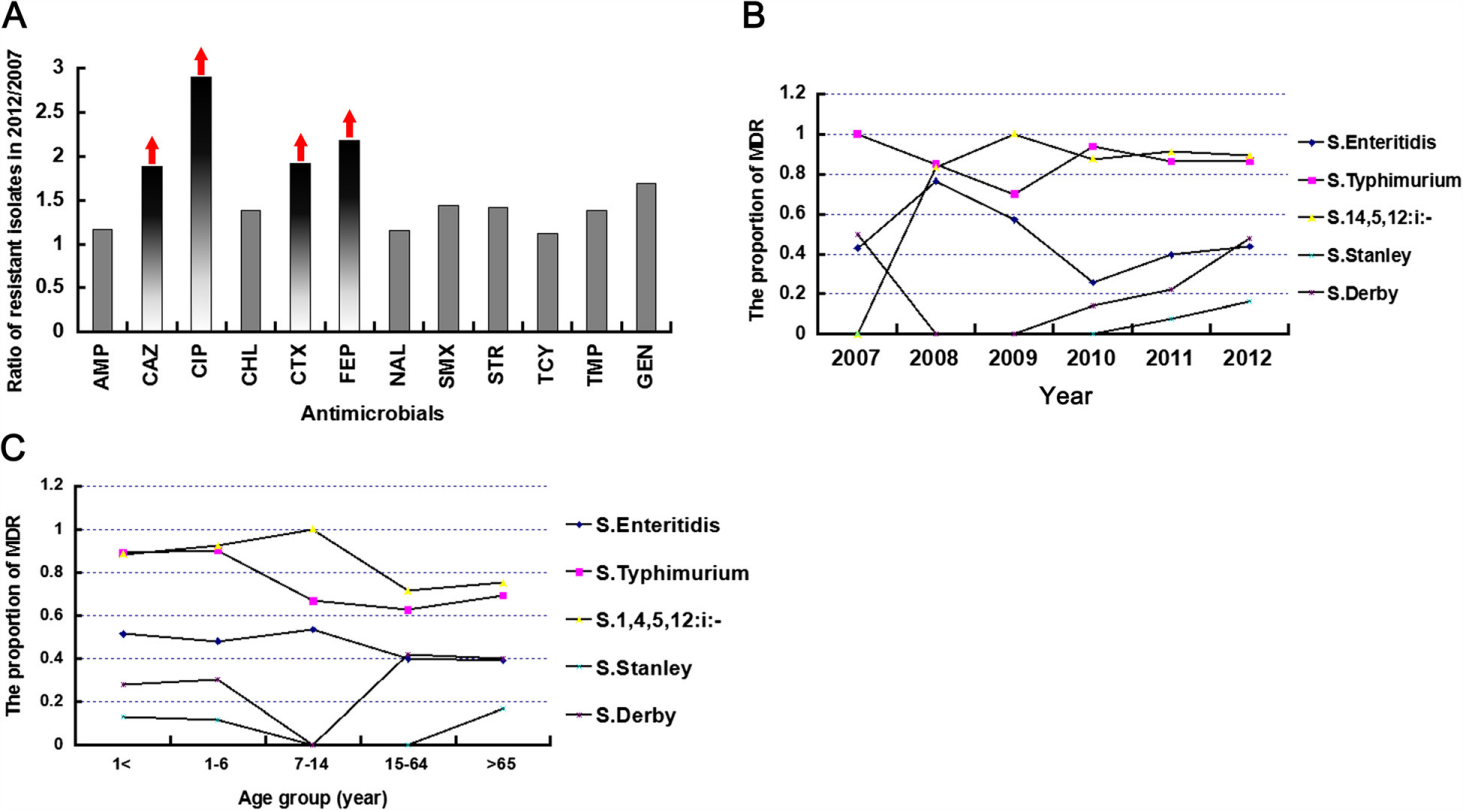
**The ratio of resistant isolates in 2007 vs 2012 (A), and the association of MDR isolates within top 5 serovars with time (B) and age (C).** The red arrows showed the dramatic upgoing of the proportion of resistant isolates among total isolates **(A)**. In panel **B** and **C**, Each data point means the proportion of MDR isolates in the present year, different serovar are indicated using different symbols as illustrated in figure.

The antimicrobial resistance profiles are shown in Additional file 1: Table S1. Of all the isolates, 63.49%, 59.97%, 59.64%, 58.78% and 49.77% (n = 1120, 1058, 1052, 1037 and 878) of the isolates were resistant to sulfamethoxazole, tetracycline, nalidixic acid, ampicillin and streptomycin, respectively (Table 2). Four of the twelve antimicrobial agents, ceftazidime, cefotaxime, cefepime and ciprofloxacin, showed high susceptibility (>90%), while the trend for increased resistance was much greater for these four agents than for the other eight drugs (20-30%) (Figure 4A, red axis). Within typhoid *Salmonella*, four of twenty-eight isolates were MDR strains, while only ESS12371 and ESS12984 were resistant to ciprofloxacin and cephalosporins (ceftazidime, cefotaxime and cefepime), respectively (Additional file 1: Table S1). In the NTS isolates, the proportion of *Salmonella* Typhimurium and *Salmonella* 4,5,12:i:- resistant to trimethoprim, tetracycline, gentamicin, chloramphenicol, cefotaxime and ciprofloxacin were significantly higher (*p <* 0.05) than *Salmonella* Enteritidis (Table 2). However, 8.05% of all isolates (n = 1764), showed a reduced susceptibility to ciprofloxacin (*Salmonella* Typhimurium [n = 60], *Salmonella* 4,5,12:i:- [n =28], *Salmonella* Enteritidis [n = 11], *Salmonella* Stanley [n = 8] and other 21 serovars [n = 35]) (Table 2), whereas 4.99% (88/1764) of them were resistant to ceftazidime, cefotaxime and cefepime at same time. Resistance to cefepime alone occurred in 9.58% of the isolates (169/1764, *Salmonella* Typhimurium [n = 69], *Salmonella* 4,5,12:i:- [n = 29], *Salmonella* Enteritidis [n = 18], *Salmonella* Stanley [n = 9] and other 24 serovars, [n = 44]) (Table 2).

**Table 2:** **Antimicrobial susceptibility among 1764 *Salmonella enterica *** **isolates from humans with infections in Guangdong province, China, during 2007 and 2012, and MDR in each of the top 11 serovars**

		**No. of isolates resistant to indicated agent at the indicated breakpoint (% resistance)**^***a***^												
***S. enterica*** **serovar**	**No. of isolates**	**AMP Ampicillin**	**CAZ Ceftazidime**	**CTX Cefotaxime**	**FEP Cefepime**	**NAL Nalidixic acid**	**CIP Ciprofloxacin**	**SMX Sulfamethoxazole**	**TMP Trimethoprim**	**TCY Tetracycline**	**GEN Gentamicin**	**STR Streptomycin**	**CHL Chloramphenicol**	**MDR**
Enteritidis	257	143(55.64)	11(4.28)	14(5.45)	18(7.01)	212(82.49)	11(4.28)	141(54.86)	23(89.49)	76(29.57)	23(8.95)	110(42.81)	10(3.89)	116(45.14)
Typhimurium	523	453(86.62)	43(82.21)	68(13.01)	69(13.19)	437(83.56)	60(11.47)	455(86.99)	337(64.44)	457(87.38)	340(65.01)	391(74.76)	381(72.85)	454(86.80)
1,4,5,12:i:-	244	220(90.16)	18(7.37)	30(12.29)	29(11.88)	182(74.59)	28(11.47)	220(90.16)	128(52.46)	228(93.44)	141(57.78)	164(67.21)	164(67.21)	218(89.34)
Stanley	167	14(8.38)	10(5.99)	11(6.58)	9(5.39)	19(11.37)	8(4.79)	35(20.96)	14(8.38)	20(11.98)	13(7.78)	25(14.97)	13(7.78)	18(10.78)
Derby	46	15(26.08)	2(4.35)	3(6.52)	3(6.52)	17(36.96)	4(8.69)	35(76.08)	14(30.44)	22(47.83)	14(30.44)	19(41.30)	16(34.78)	15(32.61)
Rissen	36	26(72.22)	3(8.33)	5(13.89)	6(16.67)	5(13.89)	1(2.78)	23(63.89)	30(83.33)	31(86.11)	7(19.44)	16(44.44)	5(13.89)	15(41.67)
Weltevreden	35	3(8.57)	2(5.71)	2(5.71)	2(5.71)	4(11.43)	1(2.86)	8(22.86)	4(11.43)	6(17.14)	3(8.57)	6(17.14)	2(5.71)	3(8.57)
Infantis	28	23(82.14)	0	3(10.71)	2(7.14)	23(82.14)	0	23(82.14)	19(67.86)	19(67.86)	4(14.28)	4(14.28)	21(75)	21(75)
Agona	24	4(16.67)	0	1(4.17)	3(12.5)	4(16.67)	3(12.5)	7(29.17)	3(12.5)	8(33.33)	4(16.67)	5(20.83)	5(20.83)	6(25)
Albany	22	21(95.45)	3(13.63)	4(18.18)	4(18.18)	20(90.9)	1(4.54)	22(100)	20(90.9)	21(95.45)	4(18.18)	7(31.81)	21(95.45)	20(90.9)
Newport	19	3(15.79)	2(10.53)	1(5.26)	0	4(21.05)	1(5.26)	5(26.31)	3(15.79)	6(31.57)	2(10.53)	5(26.31)	3(15.79)	4(21.05)
Typhi	4	1(25)	0	1(25)	0	1(25)	0	1(25)	1(25)	1(25)	1(25)	1(25)	1(25)	1(25)
Paratyphi (A + B + C)	24	5(20.83)	2(8.33)	1(4.16)	1(4.16)	5(20.83)	1(4.16)	4(16.66)	0	2(8.33)	1(4.16)	4(16.66)	0	3(12.5)
Other 114 NTS serovars	335	106(31.64)	28(8.35)	32(9.55)	23(6.86)	119(35.52)	23(6.86)	141(42.09)	106(31.06)	161(31.64)	54(16.11)	121(36.11)	72(21.49)	103(30.74)
Total (% resistance)^b^		1037(58.78)	124(7.03)	176(9.98)	169(9.58)	1052(59.64)	142(8.05)	1120(63.49)	702(39.79)	1058(59.97)	611(34.64)	878(49.77)	714(40.48)	997(56.52)

^a^The percentage means the proportion of MDR isolates within each serotype.

^b^The percentage means the proportion of MDR isolates in total 1764 isolates.

MDR occurred in 56.51% (997/1764) of all isolates. The reduced susceptibility was observed in all tested antimicrobial agents from 2007 to 2012 with few exceptions (*Salmonella* Enteritidis from 2008 to 2010 and *Salmonella* Stanley from 2007 to 2008) (Figure 4B). The proportion of MDR isolates increased yearly in *Salmonella* Typhimurium and *Salmonella* 4,5,12:i:- (>80%), while this proportion declined in *Salmonella* Enteritidis. The ACSSuT is the most prevalent MDR pattern (27.04%, 477/1764), particularly in *Salmonella* Typhimurium and *Salmonella* 4,5,12:i- (74.42%, 291/391). Two *Salmonella* Typhimurium and one *Salmonella* 4,5,12:i:- isolate(s) resistant to all twelve antimicrobial agents were obtained (marked red in Additional file 1: Table S1). Only 9.97% (176/1764) of isolates were fully susceptible to all tested agents (Table 2). The ratio of MDR between the sampling locations was comparable (46.5-71.4%) (Table 2). No obvious relationships between MDR or resistance to single antimicrobial agents and sampling area were observed within these isolates.

The age-associated stratified analysis showed severe MDR in *Salmonella* Typhimurium and *Salmonella* 4,5,12:i:- for all age groups, which was even worse in infants. Reduced susceptibility occurred in infants for the tested third-generation cephalosporin and quinolone (Figure 4C).

### Pulsed field gel electrophoresis

Among the 1179 isolates of *Salmonella* analyzed by PFGE, 513 different PFGE profiles were observed (Table 3). Each serovar corresponded to a single clade, while a few isolates could be clustered into other serovar clades (data not show). For the *Salmonella* Typhimurium and *Salmonella* 4,5,12:i:- complex clades, 286 PFGE profiles were observed. The PFGE profile, JPXX01.GD0004, was most prevalent, occurring in 21.37% (150/694) of isolates. For *Salmonella* Enteritidis, profiles JEGX01.GD0006 (n = 50, 21.55%) and JEGX01.GD0007 (n = 50, 21.55%) were the most common patterns. For *Salmonella* Stanley, the JNGX01.GD0006 and JNGX01.GD0007 profiles were most common patterns, while no predominant PFGE patterns were observed either in *Salmonella* Derby (24 PFGE profiles) or other serovars (53 PFGE profiles). The association analysis among the predominant PFGE patterns, MDR, age and geographic location showed that ACSSuT was the predominant MDR pattern in the *Salmonella* Typhimurium & S. 4,5,12:i:-complex clade, while ASSuT-Nal (nalidixic acid)and ASSu-Nal were the predominant MDR profiles in *Salmonella* Enteritidis. No dominant MDR patterns were observed in *Salmonella* Stanley, *Salmonella* Derby or other serovars (Table 3). The predominant PFGE patterns of the *Salmonella* Typhimurium & S. 4,5,12:i:-complex clade and *Salmonella* Stanley mainly prevalent in infants (<6 years). However, no obvious relationship was observed between the PFGE profiles and geographic location (Table 3).

**Table 3:** The association analysis among predominant PFGE patterns in each serovar, MDR, age and geography location

	**S. Typhimurium & S. 4,5,12:i:-**	**S. Enteritidis**	**S. Stanley**	**S. Derby**	**Others serovars**
Number of PFGE pattern (n)	286(n = 702)^a^	78(n = 232)	72(n = 150)	24(n = 28)	53(n = 77)
Predominant PFGE pattern (n, %)^b^	JPXX01.GD0004	JEGX01.GD0006	JNGX01.GD0006	None	None
	(n = 150, 21.37%)	(n = 50, 21.55%)	(n = 23, 15.33%)		
		JEGX01.GD0007	JNGX01.GD0007		
		(n = 50, 21.55%)	(n = 29, 19.33%)		
Age ≤ 6 years	141	51	44		
>6 years	9	49	8		
Location	Dongguan	Dongguan	Dongguan		
	Foshan	Foshan	Foshan		
	Guangzhou	Guangzhou	Guangzhou		
	Jiangmen	Heyuan	Jieyang		
	Jieyang	Huizhou	Maoming		
	Maoming	Jiangmen	Zhanjiang		
	Shantou	Jieyang	Zhaoqing		
	Yangjiang	Maoming	Zhongshan		
	Zhaoqing	Shaoguan	Zhuhai		
	Zhongshan	Yangjiang			
	Zhuhai	Zhongshan			
		Zhuhai			
MDR pattern (n, %)^c^	ACSSuT (99, 66.00%)	JEGX01.GD0006	JNGX01.GD0006		
		None	None		
		JEGX01.GD0007	JNGX01.GD0007		
		ASSuT-Nal (30, 30%)	None		
		ASSu-Nal (17, 17%)			

^a^n = number of isolates which PFGE data available.

^b^n = number of isolates in the predominant PFGE pattern, % = percentage in isolates with PFGE data.

^c^n = number of MDR isolates in the predominant PFGE pattern, % = percentage in predominant PFGE pattern isolates.

## Discussion

The current study indicated the serovar profiles of *Salmonella enterica* in diarrheal outpatients, and confirmed widespread MDR and reduced ciprofloxacin and third-generation cephalosporin susceptibility among NTS isolates from Guangdong province. Although typhoid *Salmonella* occurred in a few cases, most of them showed no resistance to ciprofloxacin or third-generation cephalosporins. These results were in agreement with the general knowledge of the epidemiology of salmonellosis [6].

The serovar profile data showed that *Salmonella* Typhimurium became the dominant serovar in 2008. *Salmonella* 4,5,12:i:- is a monophasic variant of *Salmonella* Typhimurium, which is very similar at the molecular level [24,25]. Therefore, the complex group of *Salmonella* Typhimurium & 4,5,12:i:- ranked first in endemic serovars of *Salmonella* in Guangdong. These data differ from those in Europe and Latin America where *Salmonella* Enteritidis is the most common serovar, followed by *Salmonella* Typhimurium [26,27]. In contrast, the Guangdong data agree with those in Africa and North America where *Salmonella* Typhimurium seems to be more common than *Salmonella* Enteritidis [28]. However, we still do not know the exact reasons for this shifting from *Salmonella* Enteritidis to *Salmonella* Typhimurium and *Salmonella* 4,5,12:i:-. As we know, *Salmonella* Typhimurium can be found in a wide range of animal species and is most commonly observed in pigs, while the most common reservoirs of *Salmonella* Enteritidis are eggs and chickens [29]. Therefore, it seems likely that the wider reservoir pattern for *Salmonella* Typhimurium and *Salmonella* 4,5,12:i:- provides them with more opportunities to come in contact with people and other reservoirs, resulting in the fast expansion of these two serovars. Another possible explanation for the increased detection of *Salmonella* Typhimurium and *Salmonella* 4,5,12:i:- in Guangdong is the updated training program for sampling and culture in 2009. After training, the total number of sampling and isolates increased significantly in 2009 (Table 1). Meanwhile, we found that both *Salmonella* Typhimurium and *Salmonella* 4,5,12:i:- affected infants, whereas *Salmonella* Enteritidis affected adults. The different eating habits of infants and adults or special host adaptations may explain the observed distribution pattern. *Salmonella* Stanley and Derby were also commonly found in Guangdong as observed in other regions of the world [26,28]. Several other serovars that are common in many countries were also found in Guangdong, such as *Salmonella* Rissen, *Salmonella* Weltevreden, *Salmonella* Infantis, *Salmonella* Agona, *Salmonella* Albany and *Salmonella* Newport [26,28]. Likely, the frequent global travel, communication and trading that occur in Guangdong favor the transmission of these serovars, as similar trends have been verified in Asia [30], Africa and Europe [31].

The antimicrobial susceptibility analyses cover antimicrobial resistance patterns in 15 of 21 cities in Guangdong where the *Salmonella* situation had been poorly characterized. Indeed, previous data about Salmonellosis in Guangdong comprise few studies [15-18]. Comparing these studies illustrates the emergence of antimicrobial resistance except for the dramatic increase of resistance to ciprofloxacin and third-generation cephalosporins, which we have summarized in Guangdong province for the first time here. A similar increase in resistance to antibiotics was observed in fish and chicken in Guangdong [32]. The observed antibiotic resistance trend in Guangdong is very similar to those described in the Guangxi [33] and Henan provinces [14] in China. The major difference is the higher ciprofloxacin resistance observed in Henan than Guangdong (54% *vs* 8.05%). Meanwhile we found resistance to both ciprofloxacin and cephalosporins in *Salmonella* Typhimurium, *Salmonella* 4,5,12:i:- and *Salmonella* Enteritidis. Ciprofloxacin is currently the recommended the first-line antibiotic by the World Health Organization for the treatment of salmonellosis [34]. Third-generation cephalosporins are considered alternative drugs for salmonellosis treatment [34]. Hence, the resistance to fluoroquinolones and third-generation cephalosporins will pose a great challenge for the effective treatment of salmonellosis. Particularly, the dramatic increase of resistance to ciprofloxacin and third-generation cephalosporins in infants is a warning signal for a prudent application of these antibiotics in clinical settings. This situation in Guangdong seems completely different from particular areas in Africa where *Salmonella* are highly susceptible to antibiotics [35]. Therefore, close surveillance of *Salmonella* and their microbial resistance patterns is essential to prevent the expansion of MDR clonal populations [36]. Furthermore, the reduced ciprofloxacin susceptibility was, in all cases, associated with nalidixic acid resistance. Therefore, the detection of nalidixic acid resistance remains a valuable test to screen for reduced ciprofloxacin susceptibility in Guangdong.

PFGE typing implicated a relatively high diversity in all serotypes, indicating that the human cases were most likely sporadic; this interpretation agrees with unrelated epidemiological background of all of the cases [37]. We found few dominant PFGE patterns in past and recent isolates of *Salmonella* Typhimurium, *Salmonella* 4,5,12:i:-, *Salmonella* Enteritidis and *Salmonella* Stanley. Thus, these common patterns seem to have been constant over time. Meanwhile, resistant patterns of each predominant PFGE pattern also differed from each other. Therefore, the different PFGE patterns perhaps came from different clonal lineage. Even so, the obtained PFGE profiles and representative strains are available for comparison with profiles obtained of *Salmonella* from other countries to track the asymptomatic carriers and identify the circulation of major clones worldwide. However, as we did not determine the genotype (such as MLST or MLVA) of the dominant serovars, such as in the *Salmonella* Typhimurium and *Salmonella* 4,5,12:i:- isolates, we do not know if these serovars belong to the same lineages that are found in other continents; therefore, it would be of crucial importance to determine the genotype to link the possible transmission pathway of each predominant serovar in future studies [38].

## Conclusion

In conclusion, *Salmonella* Typhimurium and *Salmonella* 4,5,12:i:- were the most common serovars isolated during the observation period from 2007 to 2012 in Guangdong, mainly causing salmonellosis in infants, whereas *Salmonella* Enteritidis mainly caused salmonellosis in adults. In 2008, a serovar shift from *Salmonella* Enteritidis to *Salmonella* Typhimurium became apparent. A high burden of multidrug resistant strains and an increasing incidence of quinolone and third-generation cephalosporin resistance were observed. Close surveillance of *Salmonella* and their microbial resistance patterns, using combined molecular tools, such as PFGE, multilocus sequence typing (MLST) and multiple locus VNTR analysis (MLVA), is therefore dedicate to prevent outbreaks of disease, track the potential transmission pathways and rationalize the antimicrobial therapy of salmonellosis.

## Competing interests

The authors declare that they have no financial or non-financial competing interests.

## Authors’ contributions

BK, DH, XL and ZL contributed to acquisition of data. JS and BK conducted the analysis, interpretation of data and manuscript drafting. CK contributed to the conception and design of this study and the manuscript revision. All authors read and approved the final manuscript.

## Pre-publication history

The pre-publication history for this paper can be accessed here:

http://www.biomedcentral.com/1471-2334/14/338/prepub

## Supplementary Material

Additional file 1: Table S1 The isolates collected in this study, patient information, antimicrobial susceptibility test profiles and PFGE results. The isolates resistant to all tested antimicrobials are labeled with red. Click here for file
